# The Effect of Dietary Supplementation with Resveratrol on Growth Performance, Carcass and Meat Quality, Blood Lipid Levels and Ruminal Microbiota in Fattening Goats

**DOI:** 10.3390/foods11040598

**Published:** 2022-02-18

**Authors:** Yujian Shen, Yuhang Jiang, Sanbao Zhang, Juhong Zou, Xiaotong Gao, Ying Song, Yu Zhang, Yan Hu, Yanna Huang, Qinyang Jiang

**Affiliations:** College of Animal Science and Technology, Guangxi University, 100 East University Road, Nanning 530004, China; shenyj1991@126.com (Y.S.); jiangyh1996@126.com (Y.J.); gxuzsb@126.com (S.Z.); zz898600@126.com (J.Z.); gaoxt855@163.com (X.G.); sy970526@126.com (Y.S.); zy13679430243@126.com (Y.Z.); hy2789307025@163.com (Y.H.); huangyn@gxu.edu.cn (Y.H.)

**Keywords:** resveratrol, goat, growth performance, meat quality, ruminal microbiota

## Abstract

This study investigated the effects of resveratrol (RES) supplementation on the growth performance, carcass and meat quality, blood lipid levels and ruminal bacterial microbiota of fattening goats. A total of forty castrated Nubian goats (28.25 ± 0.26 kg body weight) were randomly divided into four groups and provided with diets containing different levels of RES (0, 150, 300 and 600 mg/kg) for 120 d. The results showed that RES increased redness and intramuscular fat content, whilst reducing shear force in the *longissimus dorsi* muscle of goats (*p* < 0.05). In addition, the final weight, average daily gain, hot carcass weight, net meat weight, carcass lean percentage and eye muscle area of goats were significantly increased in the 150 mg/kg RES group compared with the other three groups, while those in the 600 mg/kg RES group significantly decreased (*p* < 0.05). RES significantly decreased serum triacylglycerol and LDL-C contents (*p* < 0.05), and increased HDL-C content and the HDL-C/TC ratio (*p* < 0.05). Supplementation with 150 mg/kg RES also increased the proportion of *Acetitomaculum* and *Moryella*, genera comprising short-chain fatty acid-producing bacteria. The present study indicated that an appropriate supplemental level of RES could improve the growth performance, neat percentage, meat quality, ruminal microbiota and serum lipid levels of fattening goats.

## 1. Introduction

Resveratrol (RES) is a variety of non-flavonoid polyphenol with a stilbene structure, which mainly occurs in grape, peanut, mulberry and *polygonum cuspidatum* [1]. It is reported that RES has a wide range of biological activities, including antioxidant activity [2], anti-inflammatory activity [3] and a regulatory role in energy metabolism [4]. Therefore, RES has the potential to be an effective dietary supplement to regulate body metabolism.

Recently, more and more scholars have performed numerous studies on the effects of RES supplementation in livestock and poultry production. In poultry, dietary RES supplementation has been shown to improve the quality of pork by changing the muscle fiber characteristics and antioxidant capacity [5,6]. Moreover, RES has been shown to act as an antioxidant to reduce the degradation in meat quality observed in broilers subjected to transport and heat stresses [7,8]. Maternal dietary RES has been found to increase average daily weight gain in piglets [9]. However, the effects of dietary RES supplementation on the growth performance and meat quality of fattening goats have not been reported.

Although RES has a variety of biological activities, its low bioavailability is in contrast to its biological function [10,11,12]. Azorín-Ortuño et al. (2011) found that only 0.5% of RES and its metabolites remained in the tissues and organs of pigs, and up to 65.1% of RES and its metabolites remained in the gastrointestinal tract after 6 h following feeding with RES (5.9 mg/kg BW) [13]. Dietary RES supplementation has been shown to significantly affect the growth of gut microbiota, increase the abundance of beneficial bacteria in rats [14] and to ameliorate obesity and liver steatosis through regulation of gut microbiota composition [11,15]. In ruminants, the rumen is one of the most important of all the gastrointestinal organs for digestion, metabolism and nutrition absorption, where feed components can be degraded, transformed and utilized by ruminal microbiota [16,17]. Therefore, it is of great significance to clarify the effects of dietary RES on rumen fermentation and rumen microbiota. However, previous studies on RES in ruminants mainly focus on its impact on methane emissions [18,19], whilst the effects of dietary RES supplementation on the rumen microbiota of fattening goats have not been reported.

This experiment was conducted to study the effects of different levels of RES on carcass traits, meat quality traits, serum lipid metabolism levels and ruminal microbial diversity in goats to clarify the application potential of RES in goat production.

## 2. Material and Methods

### 2.1. Animals and Experimental Diets

All experiments were approved by the Institutional Animal Care and Welfare Committee of Guangxi University (Nanning, China). A total of 40 (180 ± 3 d old) healthy castrated male Nubian goats with an average initial body weight of 28.25 kg were randomly divided into four groups with 10 duplicates in every group. Each goat was housed in a pen with a size of 3.0 m × 1.5 m. The goat houses were cleaned and disinfected regularly to keep the paddock ventilated, sanitary and dry. The groups were stochastically allotted to each of four dietary treatments including a control diet (basal diet) and RES containing diets (150, 300 or 600 mg/kg RES + basal diet). Ingredient compositions and nutrient contents of the basal diets for goats are presented in Table 1. The RES used in the research was purchased from Wan Fang Biotechnology CO., Ltd. (Xi’an, China) and had a purity of 98.9%. We first mixed RES into corn starch to achieve the required proportions for each diet. The goats were then fed with the corresponding RES-supplemented feed at 7:00 every morning. Goats were free to eat basal diets from 7:30 to 19:30 every day. Goats also had free access to water and a salt block during the trial period. The feeding experiment lasted for 120 d, and the daily feed intake of each goat was recorded to calculate average daily feed intake (ADFI). Individual body weight was determined before and after the trial to calculate the average daily gain (ADG) and feed conversion ratio (FCR). FCR was calculated as the ratio of total feed intake to total weight gain over the entire trial period.

### 2.2. Slaughter Surveys and Sampling

At the end of the trial, the goats were fasted for 24 h. Before slaughtering, blood samples were collected from the jugular veins of each goat using empty collection tubes. The serum samples (supernatant) were collected immediately after the blood samples had been centrifuged at 3000× *g* for 15 min at 4 °C and were then stored at −80 °C for hematological analysis.

Six goats from each group were randomly selected for slaughter, which was conducted in the large animal slaughtering room of the animal science teaching experimental base of Guangxi University. All goats were slaughtered in accordance with the agricultural industry standard of the People's Republic of China (NY/T3469-2019). Twenty-four goats were stunned, bled, skinned, gutted and then split along the midline. Hot carcass weight and net meat weight were recorded, then used to calculate dressing percentage and neat percentage. The dressing percentage refers to the percentage of carcass weight to pre-slaughter body weight, which is calculated as dressing percentage (%) = (hot carcass weight/pre-slaughter body weight) × 100. The net meat percentage refers to net meat weight as a percentage of hot carcass weight, i.e., (%) = (net meat weight/ hot carcass weight) × 100. The *longissimus dorsi* (LD) area between the 12th and 13th rib was measured using a square cm (cm^2^) grid.

Rumen fluid samples were collected from the rumen of each goat immediately after slaughter. The rumen contents were filtered through four layers of cheesecloth and collected into cryopreservation tubes, which were immediately frozen in liquid nitrogen and then stored at −80 ℃ for subsequent analysis.

### 2.3. Meat Quality and Blood Chemical Analysis

The meat quality of 24 goats was tested and analyzed according to the agricultural industry standard of the People’s Republic of China (NY/T1333-2007). A sample of LD muscle from the same area of each carcass was collected to determine pH value, color, conductivity, shear force and intramuscular fat (IMF) content.

The pH value in the LD muscle was determined at 45 min and 24 h postmortem using a pH meter (pH-STAR, Matthäus GmbH & Co. KG, Germany) equipped with a temperature compensation and calibrated using two buffers (pH 4.64 and 7.0). The meat color was detected at 45 min postmortem using a colorimeter (OPTO-Star, Matthäus GmbH & Co. KG, Germany) based on the specific parameters: L* (lightness), a* (redness) and b* (yellowness). The colorimeter was calibrated using a white tile before measurements were taken. After removing other connective tissue, the surface of the LD muscle was exposed to air for 20 min of blooming, and then measured for meat color. The conductivity of the LD muscle samples was measured using a carcass conductivity meter (LF-Star, Matthäus GmbH & Co. KG, Eckelsheim, Germany). The instrument was calibrated before measurements were taken. The probe was inserted into the sample, following the muscle fiber direction, and the conductivity values were recorded after a stabilization period. The pH, meat color and conductivity were measured repeatedly a total of 6 times, and the average values were used for further data analysis. Warner–Bratzler shear force (WBSF) was measured using a Shear Force Instrument (Mecmesin Ltd., West Sussex, UK). Briefly, the LD samples were placed in a water bath at 80 °C. After the central temperature reached 70 °C, the samples were removed and cooled to room temperature. Six cylindrical cores with a diameter of 1.27 cm were taken from each sample parallel to the direction of the muscle fibers to measure the shear force. The IMF content of the LD muscle was determined using the Soxhlet extraction method with petroleum ether extraction according to AOAC (1995) methods [20], and the results were expressed as percentages of the weight of wet muscle.

Concentrations of triglycerides (TG), total cholesterol (TC), low-density lipoprotein (LDL-C) and high-density lipoprotein (HDL-C) of serum samples were determined using automatic biochemical analyzer and direct enzymatic kits (Shanghai Kehua Bio-engineering Co., Ltd., Shanghai, China) according to the manufacturer’s instructions. Blood physiological indexes were measured using an automatic animal blood cell analyzer (Prokan Electronics Inc., Shenzhen, China).

### 2.4. Microbiota Analysis by 16S Sequencing

The genomic DNA was extracted from each goat’s rumen fluid sample (approx. 20 g) using a PowerSoil® DNA Isolation Kit (MO BIO Laboratories, Inc., Carlsbad, CA, USA) according to the manufacturer’s protocol. The concentration and purity of genomic DNA were measured using a NanoDrop 2000 instrument (Thermo Fisher Scientific, Dreieich, Germany), and the quality was evaluated via agarose gel electrophoresis analysis. The full-length bacterial 16S rRNA genes were amplified using rumen fluid genomic DNA as a template. The primers for PCR amplification were universal primers 27F (5′-AGRGTTTGATYNTGGCTCAG-3′) and 1492R (5′-TASGGHTACCTTGTTASGACTT-3′) labeled with 16 nt barcodes. PCR reactions were conducted using a 30 µL reaction including 1.5 µL of extracted genomic DNA, 3 µL of each primer (10 µM), 5 µL of KOD OneTM PCR Master Mix (Toyobo Co., Ltd., Osaka, Japan) and 10.5 µL of nuclease-free Water. The thermocycling program was as follows: an initial denaturation at 95 °C for 2 min; followed by 25 cycles of 98 °C for 10 s before annealing at 55 °C for 30 s and at 72 °C for 1.5 min; and a final extension at 72 °C for 2 min. PCR results were verified using 1.8% agarose gel electrophoresis, subsequently purified using the PureLink PCR Purification Kit (Thermo Fisher Scientific, Waltham, MA, USA) and then quantified using a Qubit 2.0 Fluorometer (Thermo Fisher Scientific, Waltham, MA, USA). The amplicon sequencing libraries were built and quality controlled. Then, qualified libraries were sequenced on PacBio Sequel II platform (Pacific Biosciences of California, Inc., Menlo Park, CA, USA).

The bioinformatics analysis of this study was carried out with the help of Biomarker Technologies Corporation, Beijing, China. The circular consensus sequencing (CCS) reads obtained from the raw reads were distributed to the matching samples on the basis of their barcodes using the LIMA software (version 1.7.0). Then, the CCS reads without primers and outside of the length range (1200–1650 bp) were removed using the Cutadapt software (version 2.7). The chimeric sequences were identified and removed to obtain the sequences of effective CCS using the UCHIME software (V4.2). USEARCH software (V10.0) was used to cluster reads with a 97% similarity cutoff to obtain the operational taxonomic unit (OTU), and the OTU was filtered when RE abundance was less than 0.005%. The RDP Classifier (version 2.2) was used to sort the feature sequences into different taxonomic groups based on the SILVA 132 reference, with a confidence threshold of 80%.

### 2.5. Statistical Analysis

Data were presented as the mean ± standard error (SEM) and statistically analyzed using a one-way analysis of variance (ANOVA) and least significant difference (LSD) test using SPSS 22.0. Statistical difference was considered significant or extremely significant at *p* < 0.05 or *p* < 0.01.

Diversity analyses of 24 samples were calculated and displayed using the QIIME (V1.8.0) and R software (V3.2.0), respectively. The measurement indexes of α diversity analyses, including Chao1, Ace, Shannon, Simpson and Coverage, were assessed using mothur (version 1.30.1). Chao1 and Ace indexes are used to measure species richness, while Shannon and Simpson indexes measure species diversity. Beta diversity analysis was computed using the Bray–Curtis distance matrix to calculate the distance between samples, and Principal coordinates analysis (PCoA) plots were used to evaluate the variation among different groups.

Characteristic differences among different treatments were analyzed using the linear discriminant analysis effect size (LEfSe). The LEfSe analysis was conducted using non-parametric factorial Kruskal–Wallis and Wilcoxon rank sum-rank tests with a linear discriminant analysis (LDA) score > 3 and *p* < 0.05. The function prediction of the ruminal microbiota was performed using phylogenetic investigation of community by reconstruction of unobserved States 2 (PICRUSt2).

## 3. Results

### 3.1. Growth and Carcass Characteristics

As shown in Table 2, compared with the control group, the final weight and ADG were significantly increased (*p* < 0.05) in the 150 mg/kg RES group and observably decreased (*p* < 0.05) in the 600 mg/kg RES group. Furthermore, the 600 mg/kg RES group had lower ADFI (*p* < 0.05) and higher FCR (*p* < 0.05) than the other three groups. Dietary 300mg/kg RES supplementation had no significant effect on the growth performance of goats.

Interestingly, dietary supplementation with 150 mg/kg RES resulted in greater hot carcass weight, net meat weight, carcass neat percentage and enlarged LD muscle area than were observed for the other supplemental levels (*p* < 0.05), while supplementation with 600 mg/kg RES had the opposite effect (Table 2). There was no significant difference in growth performance and carcass characteristics between the control and 300 mg/kg RES group. There was no noteworthy difference in dressing percentage among the four groups.

### 3.2. Meat Quality Characteristics

Dietary RES supplementation increased the a* value and IMF content and reduced the shear force in the LD muscle of goats compared with the control group (*p* < 0.05). No differences in these three indexes (*p* > 0.05) were observed among the 150 mg/kg, 300 mg/kg and 600 mg/kg RES group (Table 3). There was no significant difference in other meat quality indexes (pH_45min_, pH_24h_, L*, b* and conductivity) among the four groups (*p* > 0.05).

### 3.3. Blood Physiological and Biochemical Indexes

RES had no effect on serum TC content (*p* >  0.05) but resulted in lower (*p*  < 0.05) TG and LDL-C contents, and a greater (*p* < 0.05) HDL-C content and HDL-C/TC ratio (Table 4). In addition, there was no remarkable difference in blood physiological indexes among the four groups of finishing goats (*p*  > 0.05; Table S1).

### 3.4. Effects of Dietary RES Supplementation on Rumen Bacterial Microbiota

#### 3.4.1. Taxonomic Identification of Rumen Bacteria across Treatments

To study the effects of dietary RES supplementation on rumen fermentation and rumen microbiota, full-length 16S rRNA sequencing was used. This sequencing produced a total of 185,712 raw CCS sequences (range = 6174 to 8031, SEM = 95), obtained from 24 samples using the PacBio platform (Table S2). After read-quality filtering, a total of 165,586 high-quality effective CCS sequences were acquired, with 6629 ± 274, 6980 ± 75, 6830 ± 177 and 7159 ± 33 sequences in the control, 150 mg/kg RES, 300 mg/kg RES and 600 mg/kg RES group, respectively (Table S2). Based on a 97% sequence similarity, the average number of OTUs among all samples was 195 (range = 77 to 293, SEM = 11). The rarefaction curves on the number of OTUs showed that the sequencing depth in this study was sufficient to characterize the bacterial microbiota of the rumen fluid samples (Figure S1).

**Figure 1:**
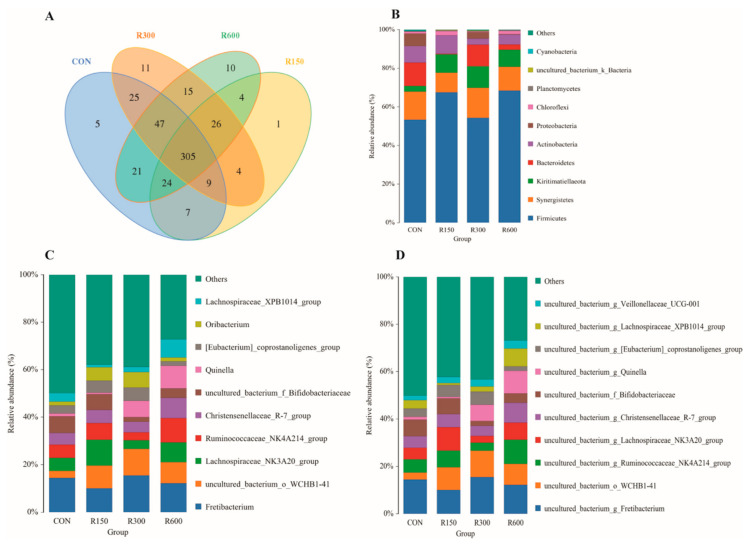
Effect of dietary supplementation with resveratrol on ruminal microbiota composition of fattening goats. (**A**) Operational taxonomic unit (OUT) Venn diagram. The number within each differently colored overlapping area is the number of OTUs shared by the overlapping groups. Non-overlapping areas indicate the number of OTUs unique to each group. The top 10 phyla (**B**), genera (**C**) and species (**D**) of the ruminal bacteria across treatments. CON, the control group; R150, the 150 mg/kg RES group; R300, the 300 mg/kg RES group; R600, the 600 mg/kg RES group.

As shown in Figure 1A, a group of 305 OTUs was shared among all four groups. The numbers of OTUs exclusive to the ruminal liquid of the goats in the control, 150 mg/kg RES, 300 mg/kg RES, and 600 mg/kg RES group were 5, 1, 11, 10, respectively, indicating that the four treatment groups had different rumen microbial species. A total of 14 phyla, 19 classes, 25 orders, 39 families, 120 genera and 144 species were detected across all the rumen fluid samples. The top 10 phyla, genera and species in terms of relative abundance among the ruminal bacteria are shown in Figure 1B–D.

#### 3.4.2. Effects of Dietary RES Supplementation on Microbial Diversity of Rumen Bacteria

**Figure 2:**
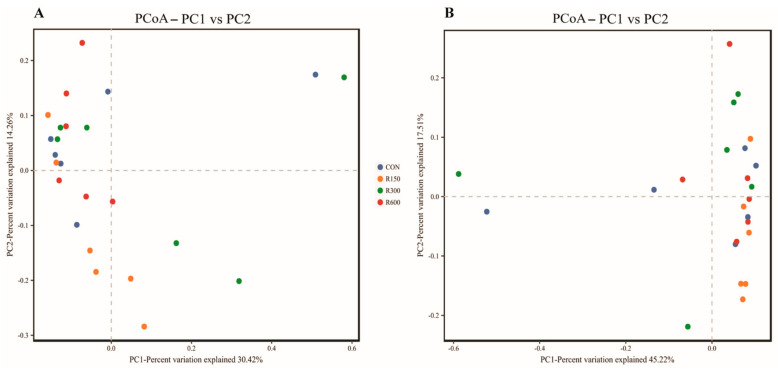
Effect of dietary supplementation with resveratrol on bacterial community structure (Principal coordinate analysis (PCoA)) in rumen fluid samples of fattening goats. PCoA based on unweighted (**A**) and weighted (**B**) Unifrac matrices.

The microbiota diversity values of the four groups exhibited no apparent differences according to α diversity indexes (ACE, Chao1, Shannon and Simpson) and the PCoA analysis based on both unweighted and weighted Unifrac distances (Figure 2), except that the 600 mg/kg RES group exhibited a higher Chao1 diversity index than the 150 mg/kg RES group (*p* < 0.05; Table 5), and some changes in the composition of the bacterial community, largely at the genus (Figure S2A) and species (Figure S2B) levels, between different groups.

#### 3.4.3. Differential Rumen Bacterial Taxa among Different Treatments

**Figure 3:**
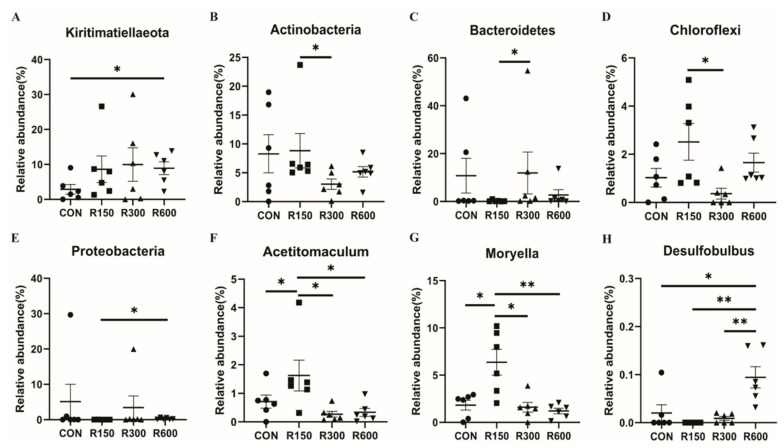
Ruminal microbiota at the phylum (**A**–**E**) and genus (**F**–**H**) level affected by dietary resveratrol supplementation in fattening goats. All values are expressed as means ± SEM, *n* = 6. * or ** represent a significant or extremely significant difference between the two groups at two terminals of the bar appearing below these characters. * *p* < 0.05, ** *p* < 0.01. CON, the control group; R150, the 150 mg/kg RES group; R300, the 300 mg/kg RES group; R600, the 600 mg/kg RES group.

We made pairwise comparisons of the relative abundances of specific microbes across the four groups. At the phylum level (Figure 3A–E), the 600 mg/kg RES group showed a higher relative abundance of *Kiritimatiellaeota* than the control group (*p* < 0.05). Compared with the 150 mg/kg RES group, the 300 mg/kg RES group had higher abundance of *Bacteroidetes* and lower abundance of *Actinobacteria* and *Chloroflexi* (*p* < 0.05). The relative abundance of *Proteobacteria* in the 600 mg/kg RES group was significantly higher than that in the 150 mg/kg RES group (*p* < 0.05). There were 23 bacterial genera significantly affected by RES supplemental levels (Table S3). Importantly, obviously higher levels of *Acetitomaculum* (Figure 3F) and *Moryella* (Figure 3G) were observed in the ruminal microbiota of the 150 mg/kg RES group than in the other three groups (*p* < 0.05). In addition, the 600 mg/kg RES group had a significantly higher relative abundance of *Desulfobulbus* than the other three groups (*p* < 0.05, Figure 3H). A total of 26 bacterial species showed significantly higher or lower relative abundance in the pairwise comparisons, mostly corresponding to the genus level (Table S4).

**Figure 4:**
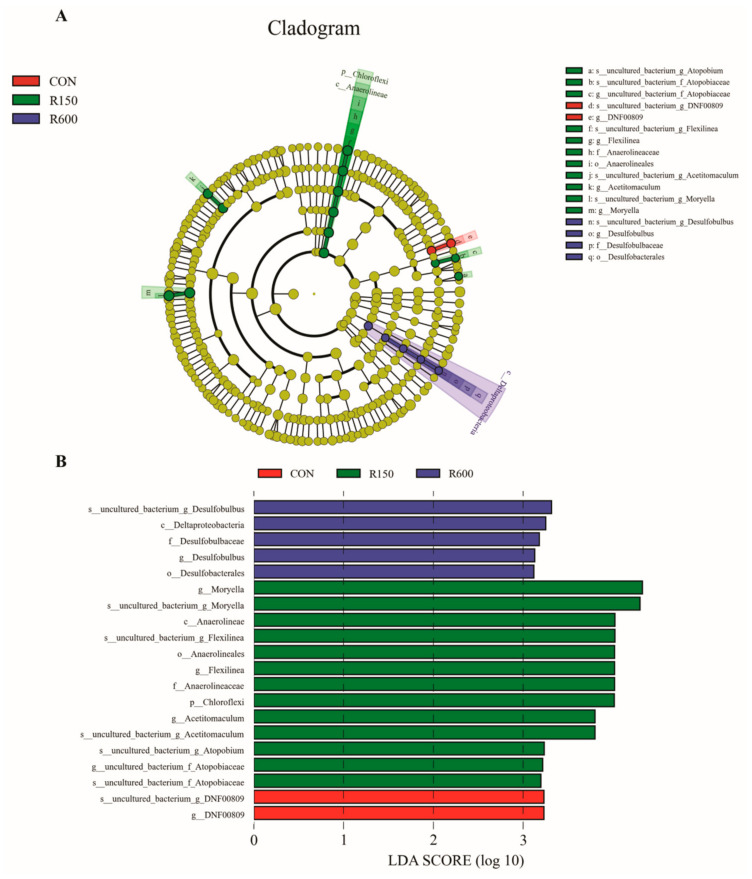
The LDA effect size (LEfSe) analysis of ruminal bacterial taxa affected by dietary resveratrol supplementation in fattening goats. (**A**) Cladogram displays significantly enriched bacterial taxa (from the class to the species level). (**B**) Bar chart displays LDA scores for different treatments. Different colors represent particular bacterial taxa that were enriched in the different groups. Significant differences are defined as *p* < 0.05 and LDA score >3.0. There were no biomarkers with statistical differences in the 300 mg/kg RES group. CON, the control group; R150, the 150 mg/kg RES group; R300, the 300 mg/kg RES group; R600, the 600 mg/kg RES group.

Significant differences in microbial species between the four groups of finishing goats were identified following LEfSe analysis (LDA = 3). Specifically, the LEfSe analysis revealed that six genera were potential biomarkers to distinguish different treatment groups (Figure 4), with one genus exclusive to the control group, four genera endemic to the 150 mg/kg RES group and one genus unique to the 600 mg/kg RES group. The control group was characterized by *g_DNF00809* and *s_uncultured_bacterium_g_DNF00809* (*p* < 0.05). The results revealed that levels of the following were significantly enriched in the 150 mg/kg RES group: *p_Chloroflexi*, *c_Anaerolineae*, *o_Anaerolineales*, *f_Anaerolineaceae*, *g_Flexilinea*, *s_uncultured_bacterium_g_Flexilinea*, *g_uncultured_bacterium_f_Atopobiaceae*, *s_uncultured_bacterium_f_Atopobiaceae, s_uncultured_bacterium_g_Atopobium*, *g_Acetitomaculum, s_uncultured_bacterium_g_Acetitomaculum*, *g_Moryella* and *s_uncultured_bacterium_g_Moryella*. The 600 mg/kg RES group, on the other hand, was marked by *c_Deltaproteobacteria, o_Desulfobacterales, f_Desulfobulbaceae, g_Desulfobulbus* and *s_uncultured_bacterium_g_Desulfobulbus*. There were no biomarkers with statistical differences in the 300 mg/kg RES group. These results indicate that different supplemental levels of RES could lead to characteristic differences in ruminal bacterial taxa.

#### 3.4.4. Functional Prediction of Rumen Microbiota among Different Treatments

**Figure 5:**
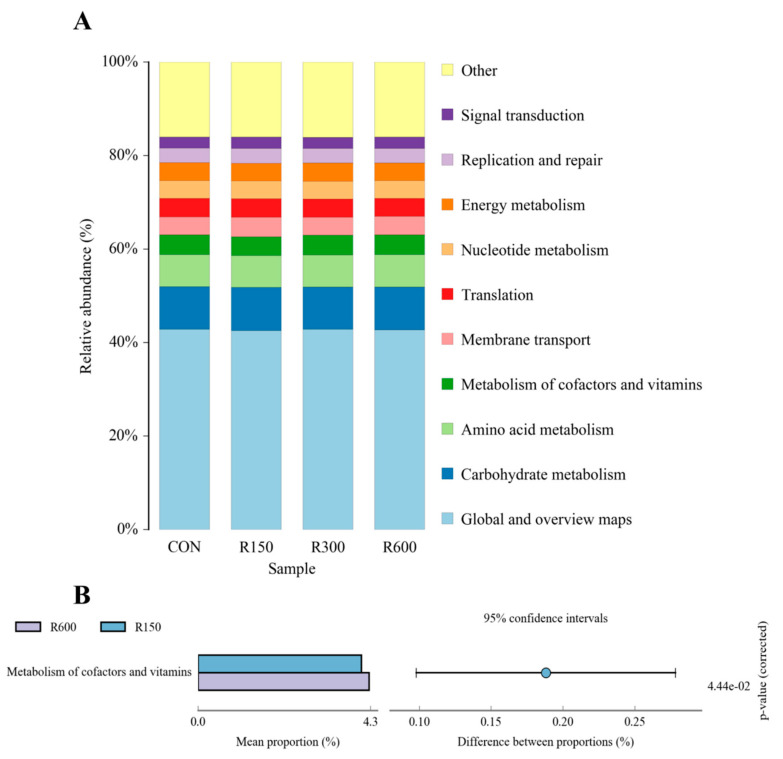
The different functions of the ruminal microbiota affected by dietary resveratrol supplementation in fattening goats. The top ten KEGG pathways (**A**) and the significant different functions (**B**) were predicted using PICRUSt2 software at level 2 across treatments. CON, the control group; R150, the 150 mg/kg RES group; R300, the 300 mg/kg RES group; R600, the 600 mg/kg RES group.

Alterations in the supposed function of the ruminal microbiota in fattening goats due to dietary RES supplementation were evaluated using the PICRUSt2 software. The KEGG Ontology (KO) abundances were mainly affected by the global and overview maps, carbohydrate metabolism and amino acid metabolism across the different groups (Figure 5A). Metabolism pathways of cofactors and vitamins were detected at significantly higher levels in the microbiota of the 600 mg/kg RES group versus the 150 mg/kg RES group (*p* < 0.05; Figure 5B), with no other significant difference in KO abundances in annotation pathways across the treatments (*p* > 0.05; Table S5).

## 4. Discussion

In order to investigate the effect of RES on fattening goats, we fed goats with different levels of RES (0, 150, 300 and 600 mg/kg) and studied the differences in growth performance, meat quality and rumen microbiota sequenced using full-length 16S rRNA sequencing. To our knowledge, this is the first research to report that adding different levels of RES could affect growth and rumen microbiota, as well as improving meat quality and plasma lipid metabolism in fattening goats.

Some studies have shown that dietary RES supplementation had no significant effect on the growth performance and carcass characteristics of finishing pigs [6,21]. However, Meng et al. (2019) found that dietary RES in sows could increase the average daily weight gains of piglets [9]. Likewise, dietary supplementation with 400 mg/kg RES improved the FCR and final body weight of broilers subjected to transport stress [7]. In this study, we observed that different supplemental levels of RES had different effects on the growth performance and carcass characteristics of goats. Treatment with 300 mg/kg RES for 120 d had no significant effect on food intake, body weight or carcass characteristics, which is consistent with some previous studies in mice and pigs [5,6,21]. Dietary RES supplementation has previously been found to have no significant effects on carcass characteristics including carcass weight, dressing percentage and LD muscle area in finishing pigs [6,21]. Consistent with this, our study found that supplementation with 300 mg/kg RES had no significant effect on these carcass traits in fattening goats. However, we also found that supplementation with 150 mg/kg RES did significantly increase the average daily gain and carcass lean percentage of goats, while dietary supplementation with 600 mg/ kg RES had the opposite effect. The lowest daily feed intake was observed in the 600 mg/kg RES group compared with the other groups, which was probably the reason for the significantly slower growth rate in the 600 mg/kg RES group. Although so far no research has reported adverse effects of RES on feed intake and growth rate, one study found that eugenol (another phenol) administered at a high level (1600 mg/d) had negative effects on fattening beef cattle [22]. High concentrations of some phenols inhibit rumen microbial growth and rumen fermentation, which are crucial for nutrient transformation in muscle tissue [23,24,25], but whether the mechanism of RES is consistent with these findings is a topic that requires further study.

Meat color is one of the most important meat quality indicators determining consumer acceptance and purchase decisions, and is related to myoglobin content, especially the a* value. Our results indicate that the a* value of the LD muscle increases with an increase in the RES supplementation level. These results were consistent with other studies on pigs [6], chickens [8] and ducks [26]. It has been reported that RES has antioxidant properties that protect mitochondrial function in muscles, prevent free radical attacks and reduce myoglobin oxidation, thus improving the color of meat [27,28]. Therefore, the increase in the a* value of the meat samples may be related to the effect of RES on antioxidant performance. IMF content is a significant index affecting meat quality, essential for the juiciness, tenderness and flavor of meat [29,30]. In this study, the IMF contents of the LD muscle were significantly increased by RES supplementation in growing-fattening goats, which is in line with findings from other studies in pigs [21] and ducks [26]. Another previous study has reported that dietary RES supplementation could significantly improve the IMF content in growing-finishing pigs, which might be connected with the promotion of intramuscular lipid anabolism and repression of intramuscular lipid catabolism [21]. The mechanism by which RES improves intramuscular fat content in fattening goats requires further study. Shear force plays an important role in the evaluation of meat tenderness, and is also one of the conventional indicators used in meat quality evaluation [31]. In this study, dietary RES supplementation significantly reduced the shear force of the LD muscle, a finding in line with a previous report on growing-finishing pigs [6]. These results reveal that dietary RES supplementation improved some meat quality indexes (a* value, IMF content and shear force) in the LD muscle of goats.

Elevated levels of total cholesterol, LDL cholesterol and triglycerides and decreased levels of HDL cholesterol in the blood are characteristic of dyslipidemia and are associated with an increased risk of cardiovascular disease [32,33,34]. Previous reports on animals and humans have indicated that RES decreased levels of triacylglycerol and LDL-C and increased the levels of HDL-C in the blood [15,32,35,36,37]. In this study, serum triacylglycerol and LDL-C levels in the RES groups were significantly decreased versus the control group. A higher HDL-C level and HDL-C/TC ratio were observed in the RES groups, consistent with the results reported by other authors [37]. HDL-C is widely referred to as good cholesterol, because it transports cholesterol currently present in body tissues and blood vessels to the liver for processing, thereby reducing the risk of developing atherosclerotic diseases, such as heart attacks and strokes [38,39]. Our results demonstrated that RES could greatly improve blood lipid profiles in fattening goats. The blood physiological indexes showed that RES had no obvious effect on the health of goats, which was consistent with the study reported by Huang et al. (2020) [5].

Up to now, the research on RES in ruminants has mainly focused on its impact on methane emissions and rumen fermentation [18,19]. Wang et al. (2020) found that RES mitigated methane production in in vitro fermentation techniques, which may be related to a decreased abundance of *Methanobrevibacter* [19]. In addition, it has been reported that dietary RES supplementation increased the proportion of *Fibrobacter succinogenes, Ruminococcus albus* and *Butyrivibrio fibrisolvens* (*p* < 0.001) while decreasing the abundance of protozoa and methanogens in the rumen fluid of sheep [18]. In this study, the ruminal microbiota was analyzed via full-length 16S rRNA gene sequencing. The results of this phase of the experiment showed that although there were no apparent differences in microbiota diversity values across the treatments, differences in ruminal microbiota abundance were observed at the phylum, genus and species level. The 150 mg/kg RES group exhibited a significant increase in the relative abundances of *Acetitomaculum* and *Moryella* compared with the other three groups in this study. The genus *Acetitomaculum* is a short-chain fatty acid-producing bacteria [40]. In addition, *Acetitomaculum* and *Moryella* both belong to the family *Lachnospiraceae*, members of which are known for their ability to synthesize short-chain fatty acids (SCFAs) through the fermentation of dietary polysaccharides [41,42,43,44], and SCFAs in turn are an important substrate for maintaining gastrointestinal epithelium and regulating the immune system and inflammatory response [45]. Consistent with these findings, Meng et al. (2019) reported that maternal dietary resveratrol increased the relative abundance of butyrate-producing bacteria in weaning piglets, and, moreover, that dietary supplementation with 600mg/kg RES increased the relative abundance of *Deltaproteobacteria*, including *Desulfobulbus* [9]. The class *Deltaproteobacteria* is the main representative of sulfate-reducing bacteria, which can counter the accumulation of toxic hydrogen sulfide gas, produced when ruminants consume large amounts of sulfate [46,47]. Elsewhere, it has been reported that hydrogen sulfide can inhibit the production of SCFAs, in particular preventing the oxidation of butyrate, therefore affecting energy acquisition, weight gain and feed efficiency [48]. The results of the present research showed that dietary RES supplementation could affect the composition of rumen microbiota in goats, and that these effects differ depending on the level of RES supplementation.

## 5. Conclusions

In this study, we found that an appropriate supplementation of RES (150 mg/kg) can ameliorate growth performance and carcass traits, improve meat quality and blood lipid metabolism levels and alter the composition of rumen microbiota in fattening goats. However, feeding high doses of RES (600 mg/kg) reduced the growth rate and meat yield of goats. Moderate use of RES can improve the production performance of fattening goats, and has potential to be developed into a goat feed additive.

## Figures and Tables

**Table 1:** Dietary ingredients and nutrient content of basal diets in fattening goats.

Ingredients, %	Content	Nutrition Levels, %	Content
Corn	16.30	Crude protein	17.00
Palm Kernel Expeller	21.00	Neutral detergent fiber	43.52
Peanut vine	12.00	Crude fat	2.72
Soybean meal	3.00	Calcium,Ca	0.65
Molasses	2.00	Total Phosphorus,TP	0.43
Manioc waste	12.00	Crude ash	7.76
Bagasse	6.00	Nitrogen free exteact	51.2
Corn skin	4.00	Acid detergent fiber	22.65
Cassava alcohol residue	9.00		
rice mill by-product	5.00		
Urea	1.20		
Dicalcium phosphate	0.40		
Limestone	1.00		
Bentonite	4.00		
Rumen protected fat	1.00		
Premix feed	2.10		
Total	100.00		

**Table 2:** Effect of dietary supplementation with resveratrol on growth performance and carcass characteristics of fattening goats.

Item	Control	150 mg/kg	300 mg/kg	600 mg/kg
Final body weight (kg)	45.09 ± 0.63 ^b^	48.90 ± 1.01 ^a^	44.67 ± 0.47 ^b^	38.04 ± 0.76 ^c^
Initial body weight (kg)	28.32 ± 0.50	28.43 ± 0.49	28.05 ± 0.45	28.20 ± 0.68
ADG (g/d)	139.75 ± 4.08 ^b^	170.59 ± 5.24 ^a^	138.44 ± 4.10 ^b^	82.06 ± 4.86 ^c^
ADFI (kg/d)	1.10 ± 0.09 ^a^	1.17 ± 0.11 ^a^	1.11 ± 0.14 ^a^	0.89 ± 0.07 ^b^
FCR (kg/kg)	7.87 ± 0.33 ^b^	6.99 ± 0.71 ^b^	7.48 ± 0.45 ^b^	9.20 ± 0.37 ^a^
Hot carcass weight (kg)	20.24 ± 0.43 ^b^	22.72 ± 1.00 ^a^	20.46 ± 0.53 ^b^	17.69 ± 0.51 ^c^
Net meat weight (kg)	7.44 ± 0.13 ^b^	8.62 ± 0.41 ^a^	7.32 ± 0.11 ^b^	5.71 ± 0.24 ^c^
Dressing percentage (%)	45.32 ± 0.98	45.77 ± 0.93	45.11 ± 1.36	46.50 ± 1.15
Neat percentage (%)	36.38 ± 0.43 ^b^	37.91 ± 0.45 a	36.21 ± 0.32 ^b^	34.20 ± 0.72 ^c^
LA (cm^2^)	13.33 ± 0.36 ^b^	15.21 ± 0.34 ^a^	13.83 ± 0.37 ^b^	11.83 ± 0.54 ^c^

ADG—average daily gain; ADFI—average daily feed intake; FCR—feed conversion ratio; LA—*longissimus dorsi* area. Values are shown as mean ± SEM. For the first five indexes, *n* = 10; for the last five indexes, *n* = 6. Values within a row followed by different lowercase letters are significantly different (*p* < 0.05). Dressing percentage (%) = (hot carcass weight/pre-slaughter body weight) ×100; Net meat percentage (%) = (net meat weight/ hot carcass weight) × 100.

**Table 3:** Effect of dietary supplementation with resveratrol on meat quality of fattening goats.

Item	Control	150 mg/kg	300 mg/kg	600 mg/kg
pH_45min_	6.46 ± 0.18	6.49 ± 0.07	6.46 ± 0.08	6.70 ± 0.07
pH_24h_	6.00 ± 0.06	5.80 ± 0.06	5.96 ± 0.17	5.86 ± 0.15
conductivity	3.04 ± 0.07	3.28 ± 0.11	3.04 ± 0.06	3.17 ± 0.06
Meat color:				
L*	33.42 ± 0.43	33.00 ± 0.74	32.30 ± 0.85	32.80 ± 0.52
a*	7.32 ± 0.22^b^	8.40 ± 0.27 ^a^	9.39 ± 0.49^a^	8.92 ± 0.28 ^a^
b*	6.20 ± 0.21	6.66 ± 0.46	6.60 ± 0.35	6.75 ± 0.19
Shear force(N)	86.00 ± 4.10 ^a^	70.16 ± 2.55 ^b^	66.50 ± 2.01^b^	63.23 ± 3.41 ^b^
IMF(%)	2.49 ± 0.10 ^b^	3.31 ± 0.34 ^a^	3.48 ± 0.17^a^	4.13 ± 0.46 ^a^

L*—brightness of color; a*—redness; b*—yellowness; IMF—intramuscular fat content, *n* = 6. Values are shown as mean ± SEM, *n* = 6. Values within a row followed by different lowercase letters are significantly different (*p* < 0.05).

**Table 4:** Effect of dietary supplementation with resveratrol on blood parameters of fattening goats.

Item	Control	150 mg/kg	300 mg/kg	600 mg/kg
TG (mmol/l)	0.31 ± 0.01 ^a^	0.27 ± 0.01 ^b^	0.26 ± 0.02 ^b^	0.20 ± 0.02 ^c^
TC (mmol/l)	2.55 ± 0.14	2.52 ± 0.14	2.58 ± 0.13	2.50 ± 0.09
HDL-C (mmol/l)	0.96 ± 0.06 ^b^	1.36 ± 0.08 ^a^	1.49 ± 0.11 ^a^	1.45 ± 0.09 ^a^
LDL-C (mmol/l)	0.63 ± 0.13 ^a^	0.21 ± 0.08 ^b^	0.22 ± 0.07 ^b^	0.25 ± 0.06 ^b^
HDL-C/TC (%)	38.33 ± 4.00 ^b^	54.29 ± 3.34 ^a^	57.89 ± 3.38 ^a^	58.28 ± 3.51 ^a^

TG—triacylglycerol; TC—total cholesterol; HDL-C—high-density lipoprotein cholesterol; LDL-C—low-density lipoprotein cholesterol. Values are shown as mean ± SEM, *n* = 6. Values within a row followed by different lowercase letters are significantly different (*p* < 0.05).

**Table 5:** Effect of dietary supplementation with resveratrol on bacterial alpha diversity indexes in rumen fluid samples of fattening goats.

Item	Control	150 mg/kg	300 mg/kg	600 mg/kg
Chao1	263.31 ± 38.20 ^ab^	230.17 ± 12.65 ^b^	218.45 ± 40.32 ^ab^	280.51 ± 15.24 ^a^
ACE	249.24 ± 27.79	228.30 ± 14.09	209.50 ± 40.54	274.90 ± 16.12
Shannon	5.08 ± 0.41	5.27 ± 0.19	4.84 ± 0.39	5.25 ± 0.37
Simpson	0.92 ± 0.023	0.93 ± 0.020	0.92 ± 0.019	0.92 ± 0.011

Values are shown as mean ± SEM, *n* = 6. Values within a row followed by different lowercase letters are significantly different (*p* < 0.05).

## Data Availability

The data presented in this study are available upon request from authors.

## Supplementary Materials

The following supporting information can be downloaded at: https://www.mdpi.com/article/10.3390/foods11040598/s1, Figure S1. Rarefaction curves on the OTU numbers of all the rumen fluid samples in fattening goats. X-axis represents the number of sequences picked randomly; Y-axis represents the OTU number based on the clustering of these sequences; individual curves represent each sample and are marked by different colors. The control group (CON) includes six samples: CON1-6; the 150 mg/kg RES group (R150) include six samples: RES7–RES12; the 300 mg/kg RES group (R300) include six samples: RES7–RES12; and the 600 mg/kg RES group (R600) include six samples: RES13-RES18. Figure S2. Relative abundances of genera(A) and species (B) in the rumen fluid samples of fattening goats are presented as heatmaps. CON, the control group; R150, the 150 mg/kg RES group; R300, the 300 mg/kg RES group; R600, the 600 mg/kg RES group. Table S1. Effect of dietary resveratrol supplementation on blood physiological index of goats. Table S2. Summary of the full-length 16S rRNA sequencing data of rumen fluid samples in fattening goats. Table S3. The 23 bacterial genera significantly affected by dietary resveratrol supplementation in fattening goats. Table S4. The 26 bacterial species significantly affected by dietary resveratrol supplementation in fattening goats. Table S5. Comparison of the appointed KEGG pathways across treatments using t-test based on PICRUSt2 software.
