# Supplementary material for: The Effect of Dietary Supplementation with Resveratrol on Growth Performance, Carcass and Meat Quality, Blood Lipid Levels and Ruminal Microbiota in Fattening Goats
Source: Foods. 2022 Feb 18;11(4):598. doi: 10.3390/foods11040598 (PMC8871332; doi:10.3390/foods11040598)
Supplement: Supplementary file 1 [file foods-11-00598-s001.zip › y/foods-1573770-supplementary/Supplementary Materials/Table S1-4 .pdf]

Supplementary Tables

Table S1. Effect of dietary resveratrol supplementation on blood physiological index of goats.

Parameters	Control	150mg/kg	300mg/kg	600mg/kg
White blood cell count (10^9/L)	15.92 ± 1.98	15.68 ± 1.40	16.75 ± 1.38	15.34 ± 1.38
Lymphocyte ratio (%)	33.74 ± 2.39	36.48 ± 4.07	36.44 ± 2.97	36.71 ± 12.43
Intermediate cell ratio (%)	13.71 ± 1.11	12.25 ± 0.56	14.30 ± 3.46	13.53 ± 0.68
Granulocyte ratio (%)	52.57 ± 2.61	51.27 ± 4.09	49.26 ± 2.41	49.76 ± 3.63
Lymphocyte count (10^9/L)	5.54 ± 0.92	6.06 ± 1.06	6.21 ± 0.80	5.74 ± 0.92
Intermediate cell count (10^9/L)	2.20 ± 0.32	1.91 ± 0.16	2.38 ± 0.24	2.03 ± 0.51
Granulocyte counts (10^9/L)	8.18 ± 0.97	7.71±0.56	8.16±0.73	7.57±0.78
Red blood cell count (10^12/L)	1.10±0.12	0.96 ± 0.09	0.87 ± 0.04	0.89 ± 0.05
Hemoglobin (g/L)	102.50 ± 4.51	94.30 ± 4.34	101.2 ± 1.87	103.6 ± 5.00
Hematocrit (%)	4.13 ± 0.44	3.62 ± 0.17	3.96 ± 0.19	3.74 ± 0.23
Mean corpuscular volume (fL)	42.41 ± 0.22	42.19 ± 0.25	42.57 ± 0.25	42.60 ± 0.29
Mean corpuscular hemoglobin (pg)	105.16 ± 9.11	117.81 ± 4.23	100.53 ± 8.41	118.46 ± 5.74
Mean corpuscular hemoglobin concentration (g/L)	2491.50 ± 241.01	2581.30 ± 112.54	2309.80 ± 209.13	2625.70 ± 135.40
Red blood Cell distribution width - SD (fL)	16.13 ± 0.41	16.13 ± 0.41	16.32 ± 0.25	16.13 ± 0.41
Red blood Cell distribution width - CV (%)	11.84 ± 0.28	12.14 ± 0.23	12.50 ± 0.20	12.27 ± 0.29

Values are shown as mean ± SEM.

Table S2. Summary of the full-length 16S rRNA sequencing data of rumen fluid samples in fattening goats.

Treat	Sample ID	Raw CCS	Clean CCS	Effective CCS	AvgLen(bp)	Effective(%)	ACE	Chao1	Simpson	Shannon	Coverage
Control	CON1	6174	5499	5420	1452	87.79	267.4269	263.2432	0.9708	6.0246	0.9859
	CON2	7970	7166	7088	1454	88.93	278.6064	285.7241	0.9221	4.9821	0.987
	CON3	7974	7179	7108	1452	89.14	224.3861	209.25	0.8562	4.1348	0.9915
	CON4	8023	7028	6789	1454	84.62	300.7059	392.2353	0.9558	5.6846	0.9804
	CON5	7151	6374	6282	1455	87.85	301.4747	312.2	0.9632	5.9768	0.9851
	CON6	7942	7343	7089	1449	89.26	122.8296	117.25	0.8409	3.6526	0.9961
150mg/kg	RES1	7702	7142	7140	1456	92.7	185.6078	190.1667	0.9542	5.2151	0.9951
	RES2	7984	7167	7130	1454	89.3	254.0507	252.3636	0.8364	4.3548	0.9888
	RES3	8004	7015	6899	1455	86.19	212.7329	203.8889	0.9618	5.5351	0.9907
	RES4	8007	6988	6864	1456	85.72	203.1614	220.25	0.9546	5.5721	0.99
	RES5	7353	6717	6707	1456	91.21	235.5426	242	0.9635	5.6109	0.9927
	RES6	7730	7141	7137	1455	92.33	278.7251	272.3333	0.9231	5.3035	0.993
300mg/kg	RES7	7706	7111	7101	1450	92.15	266.3959	278.875	0.9408	5.2027	0.9918
	RES8	8012	7257	6973	1458	87.03	90.5503	94	0.8636	3.5525	0.9969
	RES9	7969	7106	7005	1452	87.9	311.6897	313.0303	0.9637	5.8669	0.9862
	RES10	7960	7322	7313	1450	91.87	153.3864	168.5	0.9184	4.4448	0.9955
	RES11	7017	6367	6358	1456	90.61	312.8869	323.0312	0.9673	5.8419	0.9882
	RES12	7014	6286	6231	1450	88.84	122.0649	133.25	0.8687	4.1591	0.9955
600mg/kg	RES13	8013	7273	7210	1455	89.98	259.4854	255.3636	0.9526	5.6515	0.9927
	RES14	7988	7247	7224	1457	90.44	272.3299	278.25	0.9511	5.2927	0.9903
	RES15	8023	7246	7163	1445	89.28	240.0335	251.7727	0.7825	3.7387	0.9892
	RES16	8022	7271	7183	1456	89.54	344.2046	341	0.9788	6.4722	0.9886
	RES17	8031	7245	7174	1456	89.33	240.2376	248	0.906	4.8231	0.9906
	RES18	7943	7086	6998	1460	88.1	293.1216	308.6667	0.94	5.5342	0.9871

Table S3. The 23 bacterial genera significantly affected by dietary resveratrol supplementation in fattening goats.

Genus	Control (%)	150mg/kg (%)	300mg/kg (%)	600mg/kg (%)
Lachnospiraceae_NK3A20_group	5.90 ± 1.71^{ab}	11.53 ± 2.02^{a}	3.74 ± 1.19^{b}	8.12 ± 2.19^{ab}
Ruminococcaceae_NK4A214_group	5.77 ± 1.64^{ab}	7.57 ± 1.80^{ab}	3.33 ± 1.25^{b}	10.22 ± 3.00^{a}
uncultured_bacterium_f_p-2534-18B5_gut_group	4.01 ± 3.98^{ab}	0.0033 ± 0.0033^{b}	2.14 ± 2.00^{a}	0.26 ± 0.18^{a}
[Eubacterium]_coprostanoligenes_group	3.55 ± 0.90^{ab}	4.78 ± 1.02^{a}	5.95 ± 2.98^{ab}	1.87 ± 0.49^{b}
uncultured_bacterium_o_WCHB1-41	2.88 ± 1.35^{b}	8.62 ± 3.79^{ab}	9.96 ± 4.77^{ab}	8.91 ± 1.93^{a}
Lachnoclostridium_1	1.90 ± 1.31^{ab}	3.61 ± 1.11^{a}	0.99 ± 0.26^{b}	0.99 ± 0.34^{b}
Moryella	1.82 ± 0.52^{b}	6.37 ± 1.37^{Aa}	1.60 ± 0.52^{b}	1.20 ± 0.29^{Bb}
Ruminococcus_2	1.70 ± 1.34^{ab}	0.12 ± 0.071^{ab}	0.024 ± 0.024^{b}	0.73 ± 0.54^{a}
Prevotella_1	1.33 ± 1.01^{a}	0.16 ± 0.16^{b}	0.21 ± 0.10^{ab}	0.68 ± 0.59^{a}
Flexilinea	1.03 ± 0.39^{ab}	2.51 ± 0.76^{a}	0.36 ± 0.23^{b}	1.65 ± 0.40^{a}
Acetitomaculum	0.71 ± 0.23^{b}	1.63 ± 0.54^{a}	0.27 ± 0.10^{b}	0.33 ± 0.14^{b}
Lachnospiraceae_UCG-002	0.57 ± 0.17^{ab}	0.82 ± 0.12^{a}	0.47 ± 0.21^{ab}	0.44 ± 0.076^{b}
DNF00809	0.33 ± 0.22^{a}	0.087 ± 0.021^{a}	0.015 ± 0.010^{Bb}	0.17 ± 0.056^{Aa}
uncultured_bacterium_f_Christensenellaceae	0.23 ± 0.12^{a}	0.027 ± 0.016^{b}	0.046 ± 0.019^{ab}	0.053 ± 0.018^{ab}
Butyrivibrio_2	0.23 ± 0.11^{ab}	0.36 ± 0.12^{a}	0.094 ± 0.037^{b}	0.29 ± 0.052^{a}
[Eubacterium]_hallii_group	0.21 ± 0.082^{b}	0.65 ± 0.25^{a}	0.20 ± 0.068^{b}	0.23 ± 0.071^{ab}
uncultured_bacterium_o_Coriobacteriales	0.16 ± 0.089^{b}	1.10 ± 0.31^{a}	0.60 ± 0.45^{ab}	0.37 ± 0.15^{ab}
Desulfovibrio	0.15 ± 0.14^{ab}	0.015 ± 0.0094^{b}	0.16 ± 0.072^{ab}	0.17 ± 0.088^{a}
Atopobium	0.14 ± 0.043^{ab}	0.13 ± 0.049^{a}	0.040 ± 0.037^{b}	0.048 ± 0.018^{ab}
Erysipelotrichaceae_UCG-009	0.054 ± 0.034^{b}	1.11 ± 0.70^{a}	0.16 ± 0.11^{ab}	0.088 ± 0.041^{ab}
Lachnospiraceae_ND3007_group	0.036 ± 0.027^{ab}	0 ± 0^{b}	0.024 ± 0.024^{ab}	0.051 ± 0.036^{a}
uncultured_bacterium_f_Atopobiaceae	0.026 ± 0.0088^{ab}	0.11 ± 0.061^{a}	0.0025 ± 0.0025^{b}	0.021 ± 0.0086^{ab}
Desulfobulbus	0.020 ± 0.017^{b}	0 ± 0^{Bb}	0.0089 ± 0.0040^{Bb}	0.031 ± 0.010^{Aa}

Values are shown as mean ± SEM, *n* = 6.

Table S4. The 26 bacterial species significantly affected by dietary resveratrol supplementation in fattening goats

Species	Control	150mg/kg	300mg/kg	600mg/kg
uncultured_bacterium_g_Ruminococcaceae_NK4A214_group	5.77 ± 1.64^{ab}	7.57 ± 1.80^{ab}	3.33 ± 1.25^{b}	10.22 ± 3.00^{a}
uncultured_bacterium_g_Lachnospiraceae_NK3A20_group	5.28 ± 1.68^{AB}	10.31 ± 1.75^{A}	3.01 ± 0.93^{B}	7.06 ± 1.93^{AB}
uncultured_bacterium_f_p-2534-18B5_gut_group	4.01 ± 3.98^{ab}	0.0033 ± 0.0033^{b}	2.14 ± 2.00^{ab}	0.26 ± 0.18^{a}
uncultured_bacterium_g_[Eubacterium]_coprostanoligenes_group	3.55 ± 0.90^{ab}	4.78 ± 1.02^{a}	5.95 ± 2.98^{ab}	1.87 ± 0.49^{b}
uncultured_bacterium_o_WCHB1-41	2.88 ± 1.35^{b}	8.62 ± 3.79^{ab}	9.96 ± 4.77^{ab}	8.91 ± 1.83^{a}
Ruminococcus_flavefaciens	2.20 ± 1.97^{a}	0.19 ± 0.12^{ab}	0.022 ± 0.014^{b}	0.92 ± 0.88^{ab}
uncultured_bacterium_g_Lachnoclostridium_1	1.90 ± 1.31^{ab}	3.61 ± 1.11^{a}	0.99 ± 0.26^{b}	0.99 ± 0.34^{b}
uncultured_bacterium_g_Moryella	1.70 ± 0.49^{b}	5.91 ± 1.30^{Aa}	1.48 ± 0.49^{b}	1.07 ± 0.26^{Bb}
uncultured_bacterium_g_Oribacterium	1.64 ± 0.68^{ab}	4.50 ± 1.38^{a}	2.53 ± 0.78^{ab}	1.29 ± 0.30^{b}
uncultured_bacterium_g_Prevotella_1	1.33 ± 1.01^{a}	0.16 ± 0.15^{b}	0.21 ± 0.10^{ab}	0.68 ± 0.59^{a}
uncultured_bacterium_g_Flexilinea	1.03 ± 0.39^{ab}	2.51 ± 0.76^{a}	0.36 ± 0.23^{b}	1.65 ± 0.40^{a}
uncultured_bacterium_g_Acetitomaculum	0.71 ± 0.23^{b}	1.63 ± 0.54^{a}	0.27 ± 0.10^{b}	0.33 ± 0.14^{b}
uncultured_bacterium_g_Lachnospiraceae_UCG-002	0.57 ± 0.17^{ab}	0.82 ± 0.12^{a}	0.47 ± 021^{ab}	0.44 ± 0.076^{b}
Olsenella_scatoligenes	0.34 ± 0.15^{ab}	0.64 ± 0.30^{a}	0.24 ± 0.18^{ab}	0.057 ± 0.024^{b}
uncultured_bacterium_g_DNF00809	0.33 ± 0.22^{a}	0.087 ± 0.021^{a}	0.015 ± 0.010^{Bb}	0.17 ± 0.058^{Aa}
uncultured_bacterium_f_Christensenellaceae	0.23 ± 0.12^{a}	0.027 ± 0.016^{b}	0.046 ± 0.019^{ab}	0.053 ± 0.018^{ab}
uncultured_bacterium_g_Butyrivibrio_2	0.23 ± 0.11^{ab}	0.36 ± 0.12^{ab}	0.094 ± 0.037^{b}	0.29 ± 0.052^{a}
uncultured_bacterium_g_[Eubacterium]_hallii_group	0.21 ± 0.082^{b}	0.65 ± 0.25^{a}	0.20 ± 0.068^{b}	0.23 ± 0.071^{ab}
uncultured_bacterium_o_Coriobacteriales	0.16 ± 0.089^{b}	1.10 ± 0.31^{a}	0.60 ± 0.45^{ab}	0.37 ± 0.15^{ab}
uncultured_bacterium_g_Desulfovibrio	0.15 ± 0.14^{ab}	0.015 ± 0.0094^{b}	0.081 ± 0.038^{ab}	0.17 ± 0.088^{a}
uncultured_bacterium_g_Atopobium	0.076 ± 0.031^{ab}	0.12 ± 0.047^{Aa}	0.030 ± 0.030^{b}	0.024 ± 0.0076^{Bb}
uncultured_bacterium_g_Erysipelotrichaceae_UCG-009	0.054 ± 0.034^{b}	1.11 ± 0.70^{a}	0.16 ± 0.11^{ab}	0.088 ± 0.041^{ab}
uncultured_bacterium_g_Lachnospiraceae_ND3007_group	0.036 ± 0.027^{ab}	0 ± 0^{b}	0.024 ± 0.024^{ab}	0.051 ± 0.036^{a}
uncultured_bacterium_f_Atopobiaceae	0.026 ± 0.0088^{ab}	0.11 ± 0.061^{a}	0.0025 ± 0.0025^{b}	0.021 ± 0.0086^{ab}
Oribacterium_sp	0.024 ± 0.010^{B}	0.92 ± 0.37^{A}	4.90 ± 4.58^{AB}	0.28 ± 0.14^{AB}
uncultured_bacterium_g_Desulfobulbus	0.020 ± 0.017^{b}	0 ± 0^{Bb}	0.0089 ± 0.0040^{Bb}	0.094 ± 0.022^{Aa}

Values are shown as mean ± SEM, *n*=6.
