# Supplementary figures and images for: The Effect of Dietary Supplementation with Resveratrol on Growth Performance, Carcass and Meat Quality, Blood Lipid Levels and Ruminal Microbiota in Fattening Goats
Source: Foods. 2022 Feb 18;11(4):598. doi: 10.3390/foods11040598 (PMC8871332; doi:10.3390/foods11040598)

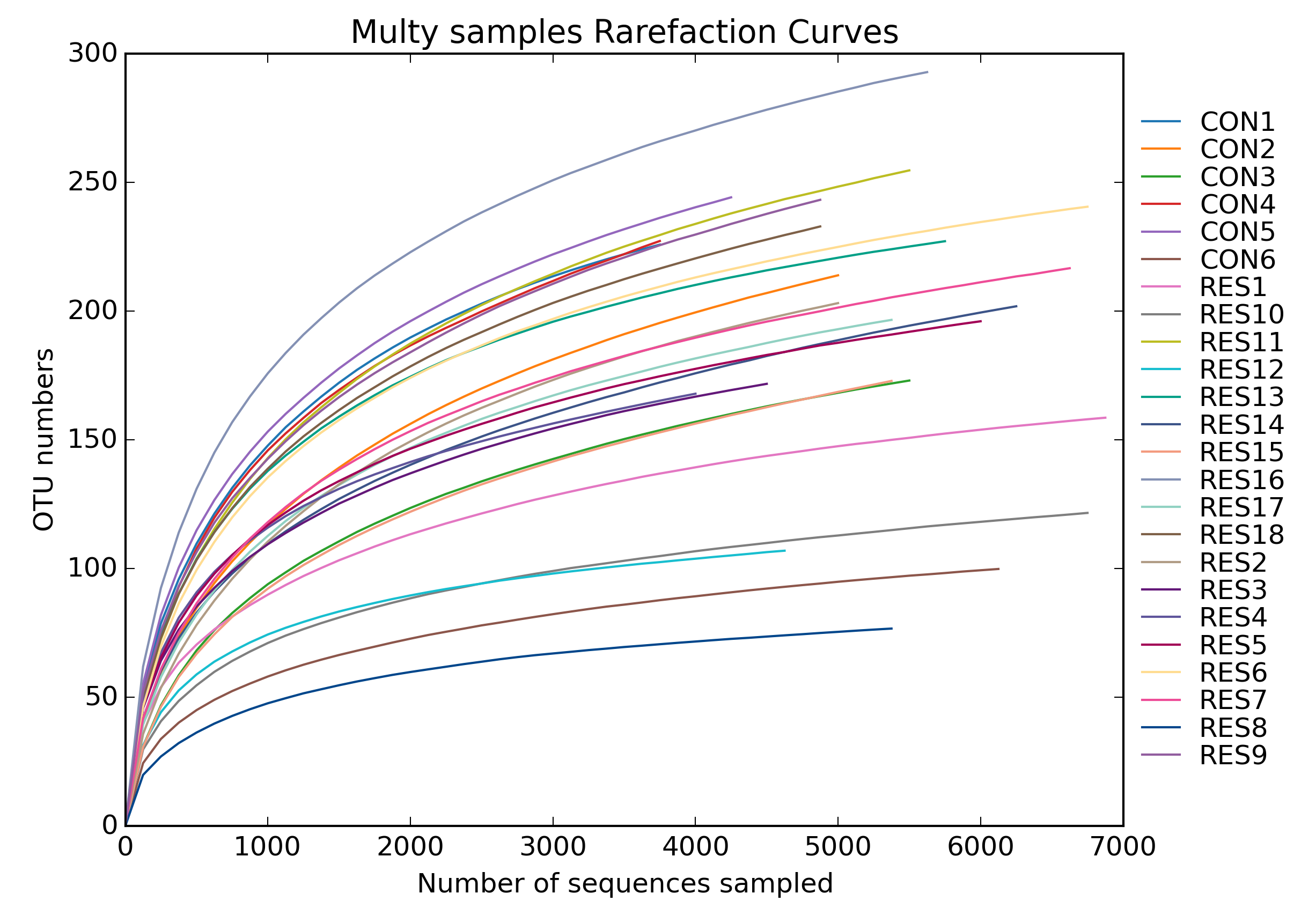

Supplement: Supplementary file 1 [file foods-11-00598-s001.zip › y/foods-1573770-supplementary/Supplementary Materials/Figure S1. Rarefaction curves on the OTUs numbers of all the rumen fluid samples in fattening goats..tif]

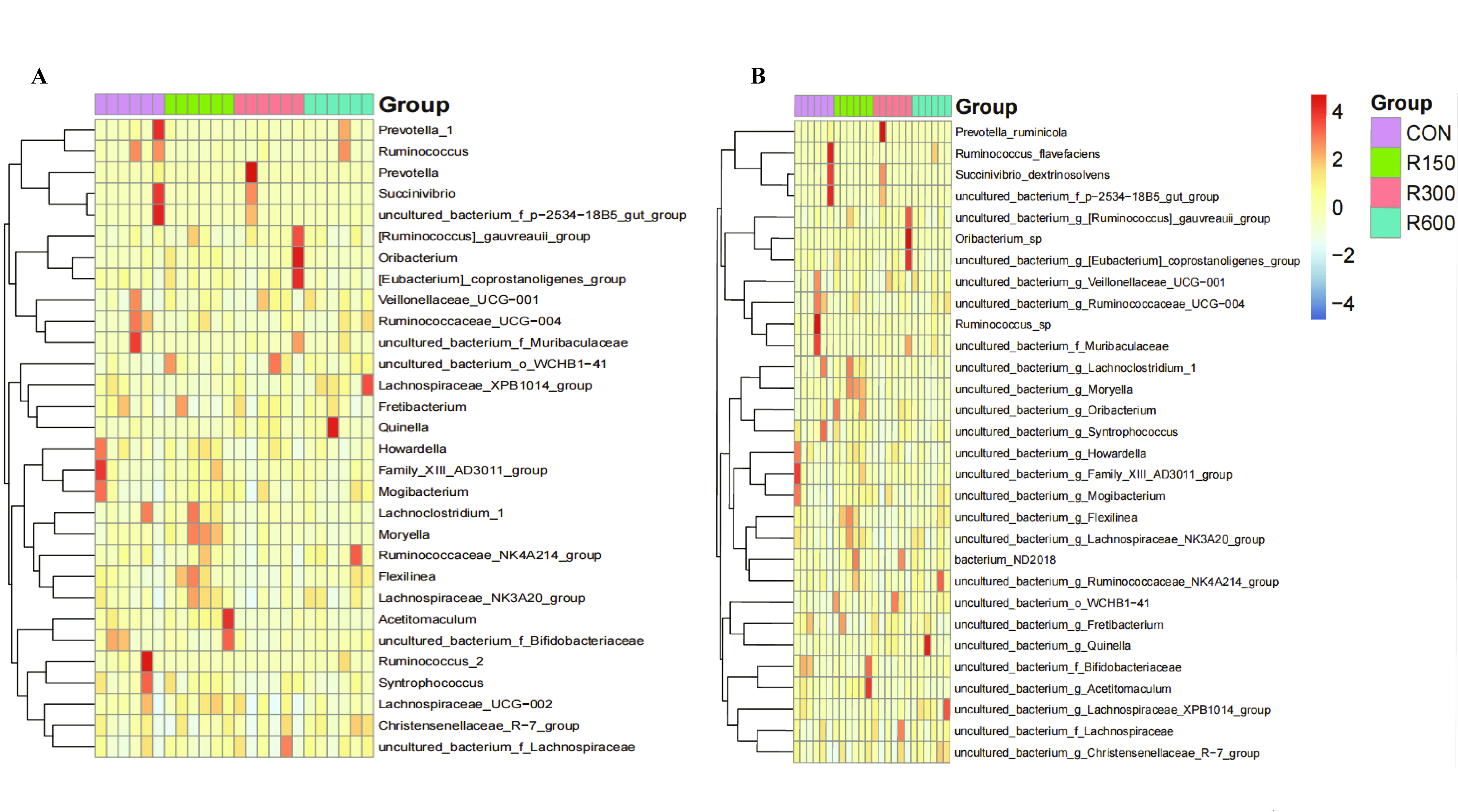

Supplement: Supplementary file 1 [file foods-11-00598-s001.zip › Figure S2. Relative abundances of genera(A) and species (B) in the rumen fluid samples of fattening goats are presented as heatmaps.tif]
